# PXR Modulates the Prostate Cancer Cell Response to Afatinib by Regulating the Expression of the Monocarboxylate Transporter SLC16A1

**DOI:** 10.3390/cancers13143635

**Published:** 2021-07-20

**Authors:** Alice Matheux, Matthieu Gassiot, Gaëlle Fromont, Fanny Leenhardt, Abdelhay Boulahtouf, Eric Fabbrizio, Candice Marchive, Aurélie Garcin, Hanane Agherbi, Eve Combès, Alexandre Evrard, Nadine Houédé, Patrick Balaguer, Céline Gongora, Litaty C. Mbatchi, Philippe Pourquier

**Affiliations:** 1 IRCM, Institut de Recherche en Cancérologie de Montpellier, INSERM U1194, Université de Montpellier, ICM, F-34298 Montpellier, France; alice.matheux@chu-dijon.fr (A.M.); matthieu.gassiot@gmail.com (M.G.); Fanny.Leenhardt@icm.unicancer.fr (F.L.); abdel.boulahtouf@inserm.fr (A.B.); eric.fabbrizio@inserm.fr (E.F.); Candice.Marchive@icm.unicancer.fr (C.M.); aurelie.garcin@inserm.fr (A.G.); hanane.agherbi@chu-nimes.fr (H.A.); eve.combes@inserm.fr (E.C.); alexandre.evrard@univ-montp1.fr (A.E.); nadine.houede@chu-nimes.fr (N.H.); patrick.balaguer@inserm.fr (P.B.); celine.gongora@inserm.fr (C.G.); litaty.mbatchi@umontpellier.fr (L.C.M.); 2 Laboratoire de Biochimie et Biologie Moléculaire, CHU Carémeau, F-30029 Nîmes, France; 3 Département de Pathologie, CHU de Tours, Université François Rabelais, Inserm UMR 1069, F-37044 Tours, France; gaelle.fromont-hankard@univ-tours.fr; 4 Laboratoire de Pharmacocinétique, Faculté de Pharmacie, Université de Montpellier, F-34090 Montpellier, France; 5 Département d’Oncologie Médicale, Institut de Cancérologie du Gard—CHU Carémeau, F-30029 Nîmes, France

**Keywords:** prostate cancer, kinase inhibitors, PXR, biomarkers, afatinib, SLC16A1

## Abstract

**Simple Summary:**

Many kinase inhibitors have been tested as potential alternatives for the treatment of castration-resistant prostate cancers. However, none of these clinical trials led to drug approval despite interesting responses. Our study reveals that genes involved in drug metabolism and their master regulator PXR (Pregnane X Receptor) could be responsible, at least in part, for these disappointing results as they can modulate tumor cell response to specific kinase inhibitors. We found that stable expression of PXR sensitized prostate cancer cells to erlotinib, dabrafenib, and afatinib, while it rendered cells resistant to dasatinib and had no effect for other inhibitors tested. We also report for the first time that sensitization to afatinib is due to an alteration in drug transport that involves the SLC16A1 monocarboxylate transporter. Together, our results further indicate that PXR might be considered as a biomarker of response to kinase inhibitors in castration-resistant prostate cancers.

**Abstract:**

Resistance to castration is a crucial issue in the treatment of metastatic prostate cancer. Kinase inhibitors (KIs) have been tested as potential alternatives, but none of them are approved yet. KIs are subject of extensive metabolism at both the hepatic and the tumor level. Here, we studied the role of PXR (Pregnane X Receptor), a master regulator of metabolism, in the resistance to KIs in a prostate cancer setting. We confirmed that PXR is expressed in prostate tumors and is more frequently detected in advanced forms of the disease. We showed that stable expression of PXR in 22Rv1 prostate cancer cells conferred a resistance to dasatinib and a higher sensitivity to erlotinib, dabrafenib, and afatinib. Higher sensitivity to afatinib was due to a ~ 2-fold increase in its intracellular accumulation and involved the SLC16A1 transporter as its pharmacological inhibition by BAY-8002 suppressed sensitization of 22Rv1 cells to afatinib and was accompanied with reduced intracellular concentration of the drug. We found that PXR could bind to the *SLC16A1* promoter and induced its transcription in the presence of PXR agonists. Together, our results suggest that PXR could be a biomarker of response to kinase inhibitors in castration-resistant prostate cancers.

## 1. Introduction

Prostate cancer (PCa) is the most commonly occurring cancer in men and remains a leading cause of death in western countries [1]. The growth of prostate cancer cells depends on the androgen axis and the transcriptional activation of the androgen receptor (AR) by its natural ligands testosterone (T) or dihydrotestosterone (DHT) [2]. This led to the approval of androgen deprivation therapy (ADT) with GnRH agonists or antagonists as the standard of care in first line treatment of locally advanced or metastatic disease. Unfortunately, most patients invariably become resistant to ADT, leading to a castration-resistant state in which tumor progression relies on the production of androgens by the adrenal glands or the tumor itself [3]. Castration-resistant prostate cancers (CRPC) may still benefit from new generation hormone therapies with AR antagonists such as enzalutamide, CYP17A1 inhibitors such as abiraterone acetate, or from taxane-based chemotherapies such as docetaxel or cabazitaxel, though survival rates do not exceed 2 years. Treatment failures are due to the development of resistant mechanisms that are either linked to AR alterations (gene amplification, point mutations, and alternative splicing) or to the activation of AR-independent signaling pathways such as the MAPK, the PI3K-AKT-mTOR, or the JAK-STAT pathways [4,5,6,7]. In the case of taxanes, specific resistance mechanisms have been described including mutations of binding sites within the microtubules or upregulation of efflux transporters such as ABCB1, ABCB4, or ABCB5 [8,9,10,11].

In the search for alternative options, a large number of clinical trials have been conducted with a wide range of anti-cancer agents, including targeted therapies such as kinase inhibitors (KIs) [12]. Although numerous studies showed that the targets of these inhibitors are deregulated in CRPC, none of the corresponding clinical trials led to drug approval. These results may be inherent to the design of these trials with a relatively small size of the cohorts, the heterogeneity of the patients’ population, and the fact that patients were not necessarily randomized based on the target alterations at the tumor level prior to enrollment. Another reason may be due to pharmacokinetics issues involving drug metabolism enzymes and transporters (DMET). Indeed, kinase inhibitors are small lipophilic molecules that are usually administered orally and are known to undergo intense metabolism at the hepatic level by cytochromes P450 such as CYP3A4 [13]. They are also known to be substrates of various ABC transporters responsible for drug efflux mechanisms resulting in reduced concentrations of the active moieties within systemic bloodstream and tumor cells. The expression of a majority of DMET genes is under the control of the nuclear receptor PXR (Pregnane X Receptor) [14]. PXR is encoded by the *NRI12* gene and can be activated by various endogenous ligands such as hormones, vitamins, or ligands from exogenous sources such as anticancer agents [15], leading to an increase in metabolism of the parent drugs and the production of pharmacologically active metabolites, or to inactive derivatives that are more readily eliminated [16]. PXR is also involved in the regulation of other cellular processes such as proliferation, metastasis, apoptosis, inflammation, and response to oxidative stress [17].

The involvement of PXR in the resistance to anticancer drugs has been extensively documented in the case of chemotherapies using various cancers cell models including osteosarcomas, hematological malignancies, as well as colon, breast, ovarian, and prostate cancer cell lines (see [18] for review). These studies showed that expression and/or activation of PXR led to a resistance to chemotherapies in most cases. This resistance was associated with a regulation of specific metabolic enzymes such as CYP3A4, conjugating enzymes from the UGT1A family, or drug transporters such as ABCB1, ABCC1, ABCC2, or ABCG2 [18]. Clinical studies also confirmed a role of PXR in the resistance to chemotherapies in patients with multiple myeloma treated with melphalan or non-small cell lung cancer patients treated with vinorelbine plus cisplatin [19,20]. In addition, a number of single nucleotide polymorphisms (SNPs) have been identified for *NR1I2* in various solid tumors, though their impact on chemotherapy efficacy was not assessed or was not significant [18].

Conversely to its role in cancer cell response to cytotoxic agents, the role of PXR in the response to kinase inhibitors was the object of fewer studies. Schellens and his colleagues were the first to demonstrate that resistance of LS180 colon cancer cells to a specific subset of kinase inhibitors (erlotinib, gefitinib, nilotinib, sorafenib, and vandetanib) relied on a PXR-mediated increase in ABCB1 expression [21]. Another study demonstrated that the B-RAF inhibitor vemurafenib could induce PXR activity in vitro and that clinical response to this inhibitor was linked to PXR-mediated regulation of drug transporters rather than CYP3A4 [22]. We found that the other B-RAF inhibitor dabrafenib could also bind and activate PXR and induce the proliferation of LS174T colon cancer cells and this effect was accompanied with an increase in the expression of PXR target genes [23]. In the case of sorafenib, activation of PXR by the phytoestrogen genistein conferred a resistance of the HepG2 hepatocellular carcinoma cell line to the drug [24], a result that was further confirmed upon PXR overexpression in the same model [25]. Interestingly, PXR is expressed in HCC tumors and is associated with poor prognosis of patients receiving sorafenib [25]. Resistance to erlotinib and dasatinib, which is observed in pancreatic ductal adenocarcinoma PACO14 cells, is also associated with an increase in the expression of *CYP3A5*, another PXR-target gene [26]. 

Regarding prostate cancer, only two studies have compared the expression levels of PXR by immunohistochemistry between cancerous and normal prostate tissues, leading to controversial results. While Chen et al. (2007) demonstrated a significant increase in PXR expression in tumors (mainly Gleason 6) compared with normal tissues, the study by Fujirama et al. showed that PXR immunoreactivity scores were significantly lower in cancerous lesions vs in benign foci [27,28]. It was also shown that PXR could modulate the sensitivity of PCa cells to chemotherapies, as downregulation of PXR in PC3 cells resulted in a sensitization to paclitaxel [27]. However, the role of PXR in the sensitivity of PCa cells to kinase inhibitors was never assessed. In the present study, we show that the detection of PXR in tumor tissues of PCa patients is correlated with advanced forms of the disease. We also report the effects of the stable expression of PXR on the sensitivity of 22Rv1 PCa cells to a series of kinase inhibitors that are approved in the clinic for the treatment of other cancers than PCa. Our results show that PXR expression increases the sensitivity to a specific subset of inhibitors including erlotinib, dabrafenib, and afatinib. They also revealed for the first time the role of the monocarboxylate transporter SLC16A1 as a potential PXR target gene that could regulate PCa cell response to afatinib. They further reinforce the notion that PXR expression levels in the tumor could be a relevant biomarker for patients’ selection in the context of future clinical trials testing kinase inhibitors in mCRPC.

## 2. Materials and Methods

### 2.1. Cell Lines

The human prostate cancer cell line 22Rv1 was obtained from the American Type Culture Collection (ATCC). Cells were cultured in RPMI 1640 medium (Thermo Fisher Scientific, Waltham, MA USA) supplemented with 10% FCS, without antibiotics at 37 °C in 5% CO2 humidified atmosphere. 22Rv1-hPXR and 22Rv1-pcDNA cells were obtained by transfection with the pcDNA3-hPXR plasmid encoding the human PXR under the CMV promoter (a kind gift from Dr. JM Pascussi, IGF, Montpellier, France) or the empty vector, and the XREM-CYP3A4-Luc+ luciferase reporter plasmid using lipofectamine 2000 (Invitrogen) according to the manufacturer’s instructions. Stable clones were isolated by limiting dilutions in the presence of 300 μg/mL of geneticin and 5 μg/mL hygromycin B. All cell lines were routinely tested for mycoplasma.

Human hepatocytes from partial hepatectomy or from human whole livers rejected for liver transplantation were purchased from Biopredic International (St Gregoire, France). Fresh plated exponentially growing hepatocytes from two different donors were received in 6-well plates and directly treated with DMSO or PXR agonists for indicated times and further processed for RNA extraction.

### 2.2. Drugs

Afatinib, cabozantinib, cobimetinib, dabrafenib, dasatinib, erlotinib, gefitinib, lapatinib, trametinib, vemurafenib, pazopanib, sorafenib, imatinib, and BAY-8002 were purchased from Selleckchem (Euromedex, Souffelweyersheim, France). Dimethysulfoxide (DMSO) and SR12813 were purchased from Sigma-Aldrich (St. Quentin Fallavier, France). 

### 2.3. Patients and Samples

The characteristics of patients and tissues are summarized in Table 1. Written informed consents were obtained from patients in accordance with the requirements of the medical ethics committee of our institutes. Clinically localized cancer samples (CLC) (*n* = 449) were obtained from patients treated by radical prostatectomy for localized PCa. Forty-eight cases of castration resistant prostate cancers (CRPC) were selected from patients treated with exclusive androgen deprivation therapy (ADT). Patients were selected when they initially responded to exclusive ADT and had post hormonal relapse tissue sample suitable for analysis. Hormonal relapse was defined as 2 consecutive rises in Prostate-Specific Antigen (PSA), with serum testosterone under castration level (50 ng/dL). Tissues were collected by transurethral resections that were performed because of lower urinary tract symptoms associated with local tumor progression. Twenty-eight cases of metastatic prostate cancer were selected from patients for whom tissues were available for analysis, either bone (*n* = 15) or lymph nodes (*n* = 13). 

### 2.4. Tissue Micro Arrays (TMA) and Immunohistochemistry

TMAs were constructed using formalin-fixed paraffin-embedded tissue samples. Original slides stained with hematoxylin-eosin were reviewed using the 2017 TNM classification and the 2014 modified “Gleason” system according to ISUP (International Society of Urological Pathology). Areas of normal tissue, tumoral tissue, or PIN were selected. For each case, 3 cores (0.6 mm diameter) were transferred from the selected areas to the recipient block, using a TMA workstation (Manual Tissue Arrayer MTA Booster, Alphelys, France). Serial 3 µm sections of the TMA blocks were used for immunohistochemistry. One section out of ten was stained with hematoxylin-eosin to check that the cores adequately represented diagnostic areas.

Slides were then deparaffinized, rehydrated, and heated in citrate buffer pH 6 for antigenic retrieval. After blocking for endogenous peroxidase with 3% hydrogen peroxide, the primary antibodies were incubated. The panel of primary antibodies included PXR (Santa Cruz clone G-11, dilution 1/50, overnight at 4 °C), CYP3A4 (Abcam EPR6202, dilution 1/500, 1 h), and the proliferation marker Ki67 (DakoCytomation, clone 39–9, 1/50, 30 min). Immunohistochemistry was performed with either the automated BenchMark XT slide stainer (Ventana Medical Systems Inc., Oro Valley, AZ, USA) for Ki67, using OptiView Detection Kit (Ventana Medical Systems Inc.) or manually (for PXR and CYP3A4) using the streptavidin-biotin-peroxydase method with diaminobenzidine as the chromogen (Kit LSAB, Dakocytomation, Glostrup, Denmark). Negative controls were obtained after omission of the primary antibody or incubation with an irrelevant antibody. Scoring of antibody staining: Ki67 was expressed as a percentage of cancer cells. PXR and CYP3A4 staining on cancer cells were expressed as positive or negative. 

Statistical analyses were carried out with StatView, version 5.0, software (Abacus Concepts, Berkeley, CA, USA). Comparison between groups was performed using the χ^2^ test for categorical data and nonparametric Mann–Whitney U test for continuous data.

### 2.5. RT-qPCR

For RNA extraction, cells were seeded in 6-well plates (10^6^ cells per well). The day after, cells were treated with indicated treatments and total RNA was extracted using *Quick RNA Miniprep* (Zymo Research, Irvine, CA, USA), according to the manufacturer’s instructions. RNA quantity and quality of samples were determined by the 260:280 nm absorbance ratios using a *Nanodrop* 2000 (Thermo Scientific, Waltham, MA, USA). Reverse transcription was performed using the SuperScript III kit (Thermo Fisher). Briefly, one μg of total RNA was added to 13.4 μL of reverse transcription mix containing 4 μL of first strand buffer 5 X, 4.5 μL of 10 mM dNTP mix, 2.5 μL of 0.1 nM dithiothreitol, 0.4 μL of oligodT primer solution, 1.5 µL of 25 mM MgCl2, and 0.5 µL of MLV-reverse transcriptase (200 U/μL) and adjusted to 25 μL final volume with RNase free water. Samples were incubated at 23 °C for 10 min, then at 42 °C for 45 min, and at 99 °C for 3 min. cDNAs were then stored at −20 °C before use.

Expression level of the different genes was evaluated by real-time quantitative PCR using a LightCycler 480 system in the presence of SYBRGreen PCR master mix 2 X (TB Green, Premix Ex Taq, Takara Bio Europe SAS, Saint-Germain-en-Laye, France) and primers used at 5 µM (final concentration). Sequences of the primers that were used are presented in Table 2. Expression levels were evaluated using the ΔΔCt method and were normalized to *GAPDH*, *HPRT*, or *RPL0*. Results are expressed as mRNA expression relative to control (untreated cells) and are the mean ± SEM of 3 independent experiments. HepaRG RNA (a kind gift from Dr. G. Chaloin, IRB, Montpellier, France) or LS-PXR RNA was used as a calibrator in qPCR reactions.

In order to screen for drug transporter genes differentially expressed between 22Rv1 cells overexpressing hPXR and control cells, we used the RT^2^ Profiler™ PCR Array Human Drug Transporters from Qiagen (Cat No./ID: PAHS-070ZF-2) and the RT^2^ SYBR Green qPCR Mastermix (Cat No./ID: 330500) for first strand cDNA synthesis reactions using the protocol recommended by the manufacturer (RT^2^ Profiler PCR Array Handbook dated 11/2018).

### 2.6. Western Blotting and Chromatin Immunoprecipitation

Protein extracts were prepared using RIPA buffer in the presence of a protease inhibitor cocktail (cOmplete™ Mini Protease Inhibitor Cocktail Tablets Roche, Sigma Aldrich, L' lsle D'Abeau Chesnes, France) or Laemmli buffer complemented by β-mercaptoethanol (5% *v*/*v*), according to standard protocols. Proteins (20 μg/lane) were separated by 10% SDS-polyacrylamide gel electrophoresis and transferred to a nitrocellulose membrane using a transfer buffer containing 20% of ethanol. Membranes were saturated by an incubation in 5% milk in PBS-Tween 0.1% and immunoblotting was performed using the following antibodies: PXR (Santa Cruz sc-48403, 1/250), CYP3A4 (Santa Cruz sc-53850, 1/250), SLC16A1 (Biotechne NBP1 56956, 1/400), GAPDH (Cell Signaling 14C10, 1/5000), and β-tubulin (Sigma-Aldrich T4026, 1/1000) and proteins were revealed by chemiluminescence using the ECL RevelBlot Plus or RevelBlot Intense (Ozyme, Saint-Cyr-l'École, France) and visualized using a G-Box (Syngene, Fisher Scientific, Illkirch, France).

Chromatin immunoprecipitations (ChIP) were performed using the ChIP-Adem-Kit and ChIP DNA Prep Adem-Kit (Ademtech, Pessac, France) and an AutoMag robot, according the manufacturer’s instructions. An amount of 25 micrograms of PFA-fixed and sheared chromatin was immunoprecipitated with 4 mg of the anti-PXR antibody (sc-48403). A non-relevant IgG antibody (Merck, 12–370) was used as negative control. The purified DNA was analyzed by qPCR using the following pairs of primers encompassing the two potential PXR binding sites in the promoter region of *SLC16A1*. For the putative site PXRBS1 in position −1045: fw1: 5′-TAGTACTTGGCCGTGGATGC and rev1: 5′-CCCCTCACTTACCCTCCCC and for the putative site PXRBS2 in position −531: fw2: 5′-TCGGTTCTACTACTGTCGCC and rev2: 5′-GGGAATGGGCAGCATTTGAA. A control ChIP was performed using the known PXR binding site in the promoter region of *CYP3A4* with the following primers: fw: 5′-AACCCAGAACCCTTGGACTC and rev: 5′-CCACCTGTGCTCTGCCTG.

### 2.7. PXR Immunofluorescence Staining

22Rv1 pcDNA and 22Rv1-hPXR cells (8000 cells/well) were plated in black-sided 96-well, flat-bottomed plates (Greiner Bio-One, Courtaboeuf, France) precoated with poly-L-ornithine. After 48 h, cells were fixed in PBS/4% paraformaldehyde for 30 min and permeabilized with PBS/0.1% Triton X-100 at 25 °C for 10 min. After blocking with PBS/1% BSA for 30 min, primary antibody against PXR (G11, Santacruz biotechnology, sc-48403) (1/200) was incubated at RT for 1 h. Then, secondary antibody (anti-mouse A488, Invitrogen, A11029, 1/400) was incubated for 45 min at 25 °C and cells were kept in the dark and stained with DAPI (1 µg/mL) for 30 min at 37 °C. Following a PBS wash, cells were analyzed using the Celigo^®^ cytometer imaging system (Nexcelom Bioscience, Lawrence, MA, USA).

### 2.8. Luciferase Assays

PXR activity was assessed in both 22Rv1 pcDNA and 22Rv1-hPXR cells using a luciferase reporter assay, as previously described [23]. Briefly, cells were seeded in 96-well white opaque flat bottom plates at 3000 cells per well in 150 μL of RPMI medium without phenol red supplemented with 5% dextran-coated charcoal-treated fetal calf serum. The day after, cells were treated with 3 µM of SR12813 for 24 h. Then, medium was removed and replaced by 50 µL of fresh medium containing 0.3 mM luciferin and luciferase activity was measured for 2 s in intact living cells after 10 min of stabilization using a MicroBetaWallac luminometer (PerkinElmer, Waltham, MA, USA).

### 2.9. Cytotoxicity Assays

Growth inhibition was measured using the standard sulforhodamine B (SRB) assay. Briefly, cells were seeded in 96-well plates (3000 cells/well). The day after, cells were incubated with indicated concentrations of the drugs for 72 h. The medium was then removed and cells were fixed with trichloroacetic acid solution (10%) and stained with a 0.4% sulforhodamine B solution in 1% acetic acid, then washed with 1% acetic acid and incubated with 10 mM Tris-HCl solution for 10 min with gentle shaking. Absorbance at 560 nm was then measured using a PHERAstar FS plate reader (BMG Labtech, Ortenberg, Germany). Percent growth was calculated in comparison with untreated cells and plotted as a function of concentrations. Results are the mean ± SEM of ≥3 independent experiments.

### 2.10. Determination of Intra- and Extra-Cellular Concentrations of Afatinib

Cells were seeded in 100 mm^2^ dishes (10^6^ per dish). The day after, the medium was removed and replaced by fresh medium without phenol red (Thermo Fisher Scientific). Cells were treated with 1 µM afatinib during 6, 24, or 48 h. At the end of the treatment, cells and supernatants were collected in separate tubes and processed in parallel. Cells were scrapped in cold PBS and centrifuged at 1500 rpm at 4 °C. Pellets were normalized for cell numbers using a separate dish treated in the same conditions and were resuspended in 1 mL of a solution of methanol/acetonitrile (50/50, *v*/*v*) and were sonicated 10 min and centrifuged at 15.000× *g* for 15 min. Then, 500 µL of supernatant was mixed with 500 µL of distilled water and centrifuged at 15.000× *g* for 15 min. Supernatant (500 µL) was then mixed with 10 µL of internal standard solution that was prepared by mixing 75 µL of (^13^C_6_)-afatinib in 5 mL of diluent buffer (50% water, 50% methanol + 0.1% formic acid). An amount of 100 µL of the former mix was then added to 200 µL of acetonitrile. Following a 15 min centrifugation at 15.000× *g*, 70 µL of the supernatant was used for analyses by HPLC-MS/MS using a Shimadzu LC-20 Prominence HPLC system (Tokyo, Japan) coupled to an AB/SCIEX API 2000 triple quadrupole mass spectrometer (Poster City, CA, USA). Chromatographic separation was performed using a XBridge C18 (100 mm × 2.1 mm, 3.5 µm) and the isocratic mobile phase conditions of 97% (*v*/*v*) methanol and water solution and 0.1 % (*v*/*v*) formic acid. The following parameters were used for afatinib: 486.178 *m*/*z* (precursor ion), 371.085 *m*/*z* (product ion), and 35 V (collision energy) for quantitation (screening transition); 486.178 *m*/*z* (precursor ion), 305.098 *m*/*z* (product ion), and 49 V (collision energy) for confirmation (confirmatory transition). For the internal standard (^13^C_6_)-afatinib, the screening transition of 492.201 *m*/*z* (precursor ion), 311.112 *m*/*z* (product ion), and 51 V (collision energy) were applied.

## 3. Results

### 3.1. PXR Expression Is More Frequently Detected in Advanced Stages of Prostate Cancers

**Figure 1:**
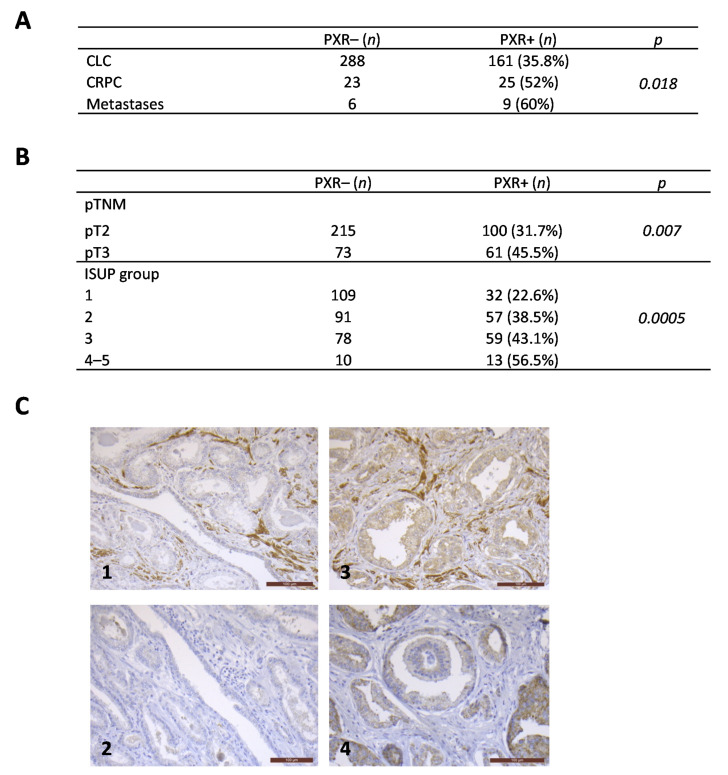
PXR expression in prostate cancer samples. (**A**) PXR expression increases with disease progression, from CLC to metastases (*p* = 0.018). (**B**) PXR staining depending on the pTNM status or on the ISUP group. PXR was more frequent in pT3 tumors when compared with pT2 (*p* = 0.007), and in tumors with high ISUP score when compared with tumors with lower ISUP score (*p* = 0.0005). (**C**) Representative immunostainings showing a positive correlation between PXR and CYP3A4 expressions (*p* = 0.0006): most tumors negative for PXR (1) were also negative for CYP3A4 (2), and most tumors that express PXR (3) also express CYP3A4 (4). PXR: Pregnane X Receptor. CLC: clinically localized cancers. CRPC: castration-resistant prostate cancers. Scale bars correspond to 100 μm.

Conflicting results regarding the PXR status in prostate cancer tissues [26,27] prompted us to evaluate its expression by immunohistochemistry using a tissue microarray including 512 samples from 449 patients with clinically localized disease (CLC), 48 patients with castration-resistant disease (CRPC), and 15 patients with metastatic disease (MET). We first validated the PXR immunostaining on paraffin-embedded pellets of 22Rv1 cells transfected with an empty vector or with a vector expressing hPXR that were used as negative and positive controls, respectively (Figure S1B). Analyses of tumor samples revealed the presence of PXR in all of the prostate cancer stages (Figure 1). PXR expression increases with disease progression and was more frequent in CRPC and PCa metastases when compared with CLC cases (*p* = 0.018) (Figure 1A). In the group of CLC, PXR staining was observed in ~36% of cases, and was more frequent in pT3 tumors (45.5% of cases) when compared with pT2 (31.7% of cases) (*p* = 0.007) (Figure 1B). In addition, the rate of PXR expressing samples increases with the ISUP score: from 22.6% of cases in PCa ISUP score 1 to 56.5% of cases in PCa ISUP score 4–5 (*p* = 0.0005) (Figure 1B). We observed a positive correlation between PXR and CYP3A4 expressions (χ2 test) (*p* = 0.0006) (Figure 1C). Moreover, PXR expression was also associated with increased proliferation (Mann–Whitney U test) (*p* = 0.003).

### 3.2. PXR Overexpression Sensitizes 22Rv1 Cells to Specific Kinase Inhibitors

**Figure 2:**
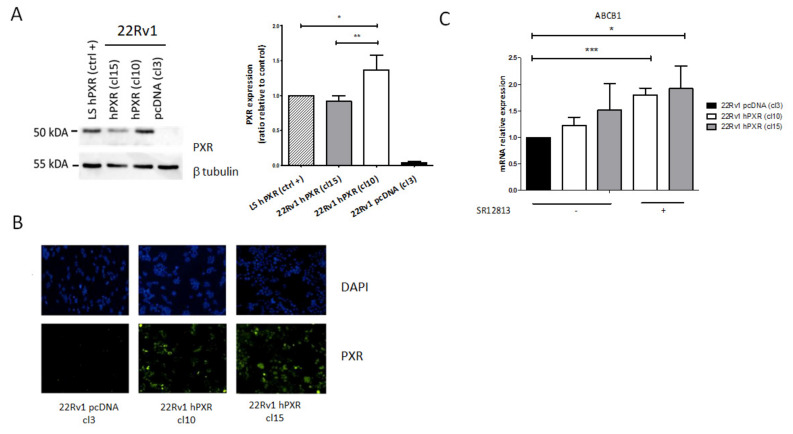
Characterization of 22Rv1 prostate cancer cells stably expressing PXR. Cells were transfected with the pcDNA3 empty vector or the pcDNA3-hPXR plasmid together with the luciferase reporter plasmid XREM-CYP3A4-Luc+ and clones stably expressing PXR (clone 10 and 15) and control clone (clone 3) were selected by limiting dilutions in the presence of antibiotics. (**A**) Evaluation of PXR protein expression by Western blotting. PXR expression was quantified using the NIH ImageJ software and normalized compared with its expression in the LS-hPXR colon cancer cell line used as a positive control. β-tubulin was used as a loading control. (**B**) Evaluation of PXR protein expression by immunofluorescence labeling. DAPI was used for nuclear staining. (**C**) Expression of PXR target gene *ABCB1* in the control clone and in clones 10 and 15 in the absence or in the presence of the PXR agonist SR12813 (3 µM, 24 h) as measured by RT-qPCR. Gene expression was normalized to the expression of *GAPDH* and plotted relative to the control clone set at 1. Results are the mean ± SEM of at least three independent experiments. Statistical analysis was carried out using Student’s *t*-test and considered statistically significant for *p* < 0.05 (*), *p* < 0.001 (**), and *p* < 0.0001 (***), respectively.

Previous studies have demonstrated the potential role of PXR in prostate cancer cell sensitivity to taxane-based chemotherapies [27], but its role in the sensitivity to targeted therapies was not investigated though many kinase inhibitors have been tested in that indication. To address this issue, we first verified the PXR status of DU145, PC3, and 22Rv1 cell lines that are usually used to mimic the advanced form of the disease and found that PXR was not expressed in these models (Figures S1A and S9). We chose the 22Rv1 human prostate carcinoma epithelial cell line to generate stable clones expressing PXR. These cells derived from the androgen-dependent CWR22 xenograft that was propagated in mice after castration-induced regression [29] and express both full length AR and its AR-v7 splicing variant and are poorly responsive to androgens. We transfected 22Rv1 cells with either an empty vector or a plasmid expressing the human PXR and the luciferase reporter gene under the control of PXR responsive elements (XREM 3A4-Luc+). We selected by limiting dilutions two clones that stably expressed PXR (clone 10 and clone 15) and a control clone (clone 3). Western blot experiments showed that PXR was expressed in both clones 10 and 15 to a similar level than in the LS-hPXR colon cancer cell line used as a positive control, whereas it was not detected in the control clone (Figure 2A and Figure S9). These data were confirmed by immunofluorescence labeling (Figure 2B). We also verified that PXR was functional in both clones 10 and 15 using a luciferase reporter assay. The results clearly showed that the activation of PXR by the reference PXR agonist SR12813 was associated with enhanced bioluminescent signal in both clones 10 and 15 (Figure S1C), demonstrating that PXR bound to its response elements present in the reporter plasmid and was transcriptionally active. The functionality of PXR was further evaluated by RT-qPCR on the PXR target gene ABCB1. The results in Figure 2C show that stable expression of PXR was associated with a significant increase in the mRNA levels of *ABCB1* in clones 10 and 15 compared with control clone, and that this expression was further enhanced by cell treatment with SR12813 (Figure 2C).

**Figure 3:**
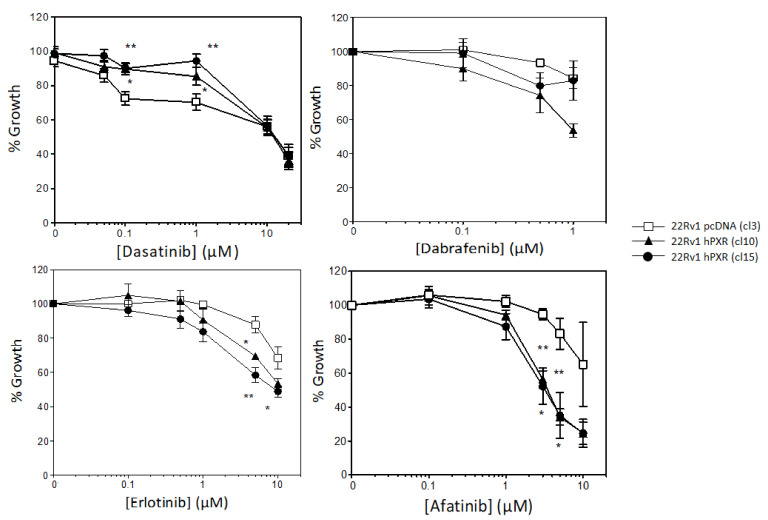
Effects of PXR stable expression on the sensitivity to kinase inhibitors. Clones 10 and 15 stably expressing PXR and the control clone (clone 3) were treated with indicated concentrations of the kinase inhibitors for 72 h and growth inhibition was measured using the standard sulforhodamine B assay. Results are plotted as a percentage of cell growth relative to untreated cells and are the mean ± SEM of at least 3 independent experiments. Statistical analysis was carried out using Student’s *t*-test and considered statistically significant for *p* < 0.05 (*) or *p* < 0.001 (**), respectively.

These clones were then used to study the effect of PXR on 22Rv1 cell sensitivity to a panel of 13 clinically approved kinase inhibitors. We showed that stable expression of PXR was associated with a resistance of 22Rv1 cells to dasatinib and a higher sensitivity to dabrafenib, erlotinib, and afatinib (Figure 3). Interestingly, this effect was particularly pronounced for the ErbB blocker afatinib. Indeed, at a concentration of 3 µM, which corresponds to its IC50 in both clones 10 and 15 expressing PXR, it barely affected cell growth of the control clone (Figure 3). 

Conversely, stable expression of PXR did not affect the sensitivity of 22Rv1 to all other kinase inhibitors that were tested (Figure S2), indicating that PXR-mediated effects on cell growth were drug-specific. 

### 3.3. PXR-Mediated Increased Sensitivity to Afatinib of 22Rv1 Cells Is Associated with Enhanced Intracellular Concentration of the Drug

**Figure 4:**
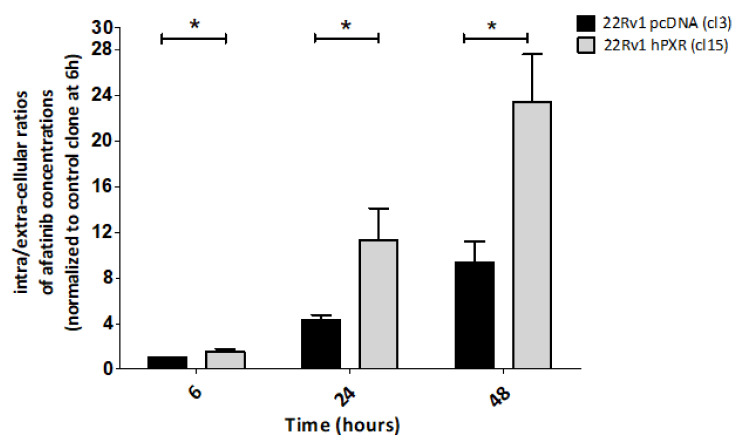
Effects of stable expression of PXR on the intracellular accumulation of afatinib. 22Rv1 cells stably expressing PXR (clone 15) and control cells (clone 3) were treated with 1 µM afatinib for 6, 24, and 48 h and concentrations of afatinib were measured using mass spectrometry (see Materials and Methods section). Results are expressed as intracellular/extracellular ratios of drug concentrations normalized to control clone that was set at one for the 6 h time point and are the mean ± SEM of 3 independent experiments. (*) *p* < 0.05 as evaluated by Student’s *t*-test.

Because afatinib was identified as a potential substrate of ABCB1 and ABCG2 transporters [30], we first investigated whether PXR-mediated sensitization of 22Rv1 cells to afatinib was accompanied with a change in its transport. For this purpose, 22Rv1-hPXR clone 15 and control clone were incubated with 1µM afatinib and intracellular concentrations of the drug as well as concentrations in the culture supernatants and were measured at 6, 24, and 48 h following treatment, using a mass spectrometry approach according to the protocol detailed in the Materials and Methods section. The ratios between intracellular and extracellular concentrations of afatinib in the control clone and in clone 15 stably expressing hPXR were calculated as a function of time and were normalized to the one obtained for the control clone at 6 h (set at 1). The results clearly show an increase in intracellular concentrations of afatinib as a function of time in clone 15 (Figure 4). 

The results also evidenced a significant increase in the ratios (of approximately three-fold) in clone 15 expressing hPXR compared with the control clone at each time point. A similar increase in afatinib intracellular concentration was also observed in 22Rv1-hPXR clone 10 (Figure S3A). We verified that these effects were not due to a degradation of afatinib in culture medium, as incubation of the drug in cell-free medium up to 72 h did not lead to a change in its concentrations (Figure S3B). Together, these results confirmed that stable expression of PXR in 22Rv1 cells is associated with an increased intracellular concentration of the inhibitor. Because afatinib metabolism is minimal [31], they further suggest that PXR-mediated increase in intracellular concentration is related to a modification in drug transport. 

### 3.4. SLC16A1 Plays a Role in the Transport of Afatinib in 22Rv1 Cells Expressing PXR

**Figure 5:**
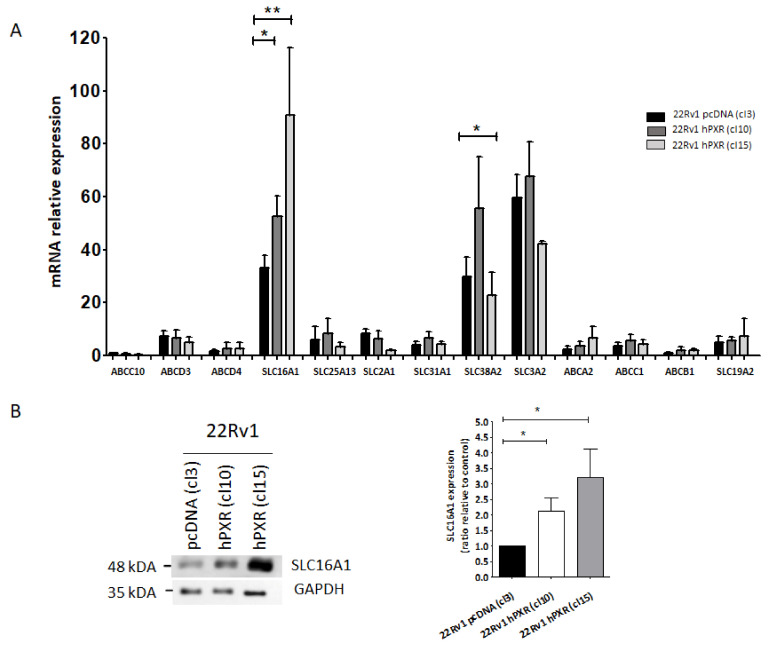
Effects of PXR on the expression of drug transporters. (**A**) Comparison of the expression levels of a set of 13 genes that were identified using the RT^2^ Profiler™ PCR Array Human Drug Transporters from Qiagen between 22Rv1 clones 10 and 15 stably expressing PXR and control clone (clone 3) as measured by RT-qPCR. Gene expression was normalized to the expression of *HPRT* and plotted relative to the expression of the *ABCC10* gene expression in the control clone (set at 1). Results are the mean ± SEM of at least three independent experiments. (**B**) Immunoblot analysis of SLC16A1 expression in control clone and clones 10 and 15. GAPDH was used as a loading control and quantification was performed using the Image J software. Results are plotted as ratios of SLC16A1 expression relative to control clone and are the mean ± SEM of three independent blots. Statistical analyses were carried out using Student’s *t*-test and considered statistically significant for *p* < 0.05 (*) or *p* < 0.001 (**), respectively.

In order to identify which potential transporter might be involved in PXR-mediated sensitization of 22Rv1 cells to afatinib, we used the RT^2^ Profiler™ PCR Array from Qiagen to evaluate the expression of the 86 main transporter genes. We measured the expression level of these genes in the control clone and in clone 10 stably expressing hPXR, paying a particular attention to influx transporters (Figure S4). Some of these transporters were expressed at very low levels (with Cycle threshold, Ct > 30) and were excluded from the analyses, as it is unlikely that they could participate to afatinib transport. Using an independent set of validated primers (Table 2), the expression of 13 transporters (Ct < 30) was re-assessed in control clone and both clones expressing hPXR. The results of Figure 5A show that *SLC16A1*, *SLC38A2* and *SLC3A2* were highly expressed in all clones, whereas other transporters were expressed at low levels.

No differential expression was noticed between control clone and both clones expressing hPXR with the exception of the SLC16A1 transporter that showed a 1.5- to 2.5-fold increase in expression at the mRNA levels in both clones 10 and 15, when compared with the control clone. Accordingly, Western blot analyses showed that SLC16A1 protein level was enhanced by two- to three-fold in 22Rv1 cells stably expressing PXR compared with control cells (Figure 5B and Figure S9), while it was not detected in DU145 and PC3 cells not expressing PXR (Figures S1A and S9). Taken together, our results suggested that SLC16A1 could be a good candidate responsible for the influx of afatinib in 22Rv1 cells stably expressing hPXR.

### 3.5. SLC16A1 Inhibition Abrogates PXR-Mediated Sensitization of 22Rv1 hPXR Cells to Afatinib

To confirm the functional implication of SLC16A1 in the influx of afatinib, we used the BAY-8002 derivative, a specific inhibitor of this influx transporter [32]. We first studied the cytotoxicity profile of BAY-8002 in 22Rv1 cells and found that concentrations of up to 1 mM did not have a significant impact on cell growth (Figure S5). We then compared the effects of afatinib on the growth of 22Rv1 clones expressing hPXR and on the control clone in the absence and in the presence of 10 µM BAY-8002.

**Figure 6:**
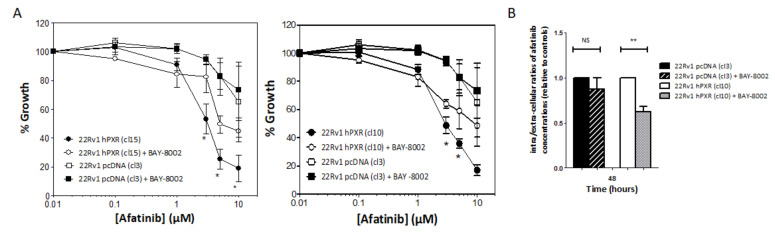
Inhibition of SLC16A1 alters PXR-mediated response of 22Rv1cells to afatinib. (**A**) Effects of the SLC16A1 inhibitor BAY-8002 on the sensitivity of the control clone compared with 22Rv1 clones 15 (left panel) and 10 (right panel) stably expressing PXR to afatinib. Cells were treated in the absence or in the presence of 10 µM BAY-8002 and indicated concentrations of afatinib for 72 h and cell growth was evaluated using sulforhodamine B. Results are plotted as percentage of cell growth relative to untreated cells. (**B**) Effects of BAY-8002 on intracellular accumulation of afatinib. Control clone and 22Rv1 clone 10 were treated with 1 µM afatinib in the absence or in the presence of 10 µM BAY-8002 for 48 h and afatinib concentrations were measured using mass spectrometry as detailed in the Materials and Methods section. The results are expressed as intracellular/extracellular ratios of afatinib concentrations and normalized to untreated clones. Results are the mean ± SEM of at least 3 independent experiments. Statistical analysis was carried out using Student’s *t*-test and considered statistically significant for *p* < 0.05 (*) or *p* < 0.001 (**), respectively.

The presence of BAY-8002 could partially prevent the sensitization of 22Rv1 cells stably expressing PXR to afatinib, whereas it had no effect in the control clone (Figure 6A). Of note, this significant effect was only observed when high concentrations of afatinib were used. We then tested whether SLC16A1 inhibition was associated with a change in afatinib concentrations. We found that 22Rv1-hPXR clone 10 had a reduced accumulation of afatinib when cells were co-treated with BAY-8002 for 48 h, whereas inhibition of SLC16A1 did not affect the ratio of extracellular to intracellular concentration of the inhibitor in the control clone (Figure 6B). Taken together, these results showed for the first time the functional role of the SLC16A1 influx transporter in PXR-mediated prostate cancer cell response to afatinib.

### 3.6. SLC16A1 Is a PXR Target Gene

**Figure 7:**
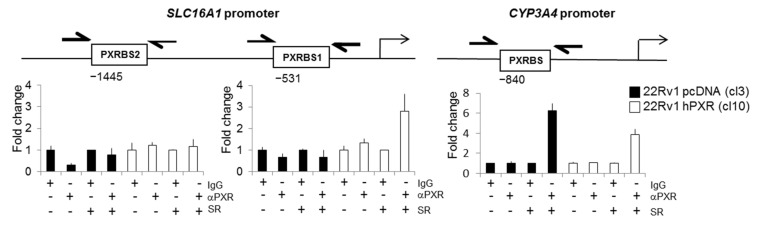
Binding of PXR in *SLC16A1* promoter by ChIP assay. Chromatin immunoprecipitation was performed using anti-PXR antibody or an irrelevant antibody in 22Rv1 control clone 3 or clone 10 stably expressing PXR, in the absence or in the presence of SR12813 (6 µM, 48 h) according to the Materials and Methods section. Purified chromatin was subjected to PCR reactions using primers encompassing each putative PXR binding site in the *SLC16A1* promoter (PXRBS1 and PXRBS2) or in the *CYP3A4* promoter. Results are expressed as a fold change of binding events relative to the results obtained for the irrelevant IgG.

Because many drug transporters are regulated by PXR, we investigated whether SLC16A1 could also be a target of this nuclear transporter. We checked whether a PXR binding site was present in the promoter of *SLC16A1* using the ALGGEN research tools (http://alggen.lsi.upc.es/ accessed on 3 September 2020). Results of Figure 7 indicated the presence of two potential consensus DNA binding motifs for PXR at -1445 bp (PXRBS1) and -531 bp (PXRBS2) upstream of the transcription starting site of the *SLC16A1* gene promoter. 

Next, we used a chromatin immunoprecipitation (ChIP) assay to investigate whether PXR could bind to these potential response elements. In the absence of SR12813, no significant binding of PXR was observed neither to the *SLC16A1* nor to the *CYP3A4* gene promoter region used as a control, regardless of the expression of PXR (Figure 7). Conversely, cell treatment with SR12813 (6 µM, 48 h) increased up to ~three-fold the recruitment of PXR to the PXRBS1 binding site in clone 10 stably expressing PXR. A similar increase in PXR binding to the promoter region of *CYP3A4* was also observed in both clones 10 and control clone 3, with 3.88- and 6.25-fold variation, respectively. Similar results were obtained using the 22Rv1 clone 15 (Figure S7). Unexpectedly, binding of PXR to the SLC16A1 promoter was not accompanied with a significant increase in SCL16A1 mRNA levels following treatment with SR12813, though a trend was observed for clone 10 (~1.5-fold) at 48 h and was of the same order of magnitude as the one that was observed for the PXR target gene ABCB1 (Figure 2C). Nevertheless, using primary hepatocytes as a more physiological model, a similar increase in SLC16A1 mRNA levels of ~four-fold was also observed following treatment with the PXR agonist dabrafenib (Figure S6), further suggesting that *SLC16A1* is a PXR target gene. 

## 4. Discussion

Numerous clinical trials have been conducted with kinase inhibitors in order to propose alternative treatments for patients with metastatic castration-resistant prostate cancers. Despite some interesting responses, none of these molecules are clinically approved in that setting yet. It is known that most of the kinase inhibitors are heavily metabolized both at the hepatic and the tumor level, but the role of metabolism in prostate tumor response to KIs has not been investigated in details, in particular the role of the pregnane X receptor (PXR) that regulates the expression of a large set of DMET genes involved in the metabolism of a large panel of xenobiotics [18].

Using tissue microarrays including more than 500 patients’ samples we could confirm the endogenous expression of PXR in prostate tumors that were previously reported [27]. We found that PXR expression was more frequently detected in samples from CRPC tumors compared with clinically localized tumors. Our results differ from a previous study showing a higher rate of PXR positivity in benign tissues compared with tumor tissues [28], but are in accordance with the study by Chen et al. performed in 124 cancerous prostate tissues in which PXR immunoreactivity increased until Gleason 6 and remained elevated for higher Gleason scores [27]. Although PXR is involved in androgen homeostasis and can regulate the proliferation of androgen-dependent prostate cancer cells [33], its physiological roles in prostate tissue and in prostate cancer onset are still unknown. Conversely, the role of PXR in prostate cancer response to chemotherapies is clearly demonstrated as PXR agonist SR12813 confers a resistance to PC-3 cells to Taxol and vinblastine, whereas PXR downregulation results in higher sensitivity to these tubulin poisons [27].

Here, we show that stable expression of PXR altered prostate cancer cell response to a specific subset of kinase inhibitors. While the expression of PXR did not affect 22Rv1 cell response to the majority of the kinase inhibitors that we tested, it conferred a resistance to dasatinib and sensitized cells to dabrafenib, erlotinib, and afatinib—this sensitization being much more pronounced for the ErbB blocker afatinib. These results highlight the role of PXR in prostate cancer cell response to targeted therapies and also reveal drug-specific effects that might be inherent to the chemical properties of the inhibitor itself and/or a difference in the metabolic pathways that are triggered in cancer cells. 

In the case of dasatinib, resistance of 22Rv1-hPXR cells may be due to enhanced elimination of the drug via the formation of inactive metabolites, as it is primarily metabolized by cytochrome P450 CYP3A4 and also because it is a substrate of the flavin-containing monooxygenase 3 (FMO3) and UDP-glucuronosyltransferases (UGTs) [13]. This is in line with in vivo data showing that dasatinib exposure could be enhanced by ketoconazole co-treatment, whereas it was reduced by PXR-mediated activation of *CYP3A4* expression by rifampicin [13]. Resistance to dasatinib could also be due to drug efflux mechanisms since dasatinib is a substrate of both ABCB1 and ABCG2 efflux transporters which were previously shown to reduce its intracellular concentration in vitro [34].

Conversely to dasatinib, higher sensitivity of 22Rv1 cells stably expressing hPXR to the EGFR inhibitor erlotinib and to the B-RAF inhibitor dabrafenib may simply reflect that metabolization of these kinase inhibitors by cytochromes (CYP3A4, 3A5, and 1A1 for erlotinib; CYP3A4 and CYP2C8 for dabrafenib) led to the formation of active metabolites, including OSI-420 for erlotinib [35] and hydroxy-dabrafenib, which contributes to the main pharmacological activity of dabrafenib [36]. Erlotinib and dabrafenib are substrates of both ABCB1 and ABCG2 efflux transporters in vitro [37,38], which may also contribute to the differences in cell response that were observed.

Interestingly, our results showed that stable expression of PXR significantly enhanced the sensitivity of 22Rv1 PCa cells to the ErbB family blocker afatinib and that this effect was due to a time-dependent increase in intracellular concentration of the drug. Afatinib is known to be poorly metabolized [31]. It is a substrate for ABCB1 and ABCG2 efflux pumps [30] and it inhibits their activities with IC_50_ concentrations below 3 µM [39]. It is not a substrate for influx transporters from the SLC22A family or OATP family such as SLCO1B1, SLCO1B3, or SLCO2B1, but can inhibit the activity of OATP2B1 (IC_50_ of ~6 µM) and of other influx transporters such as SLCO1B1, 1B3, SLC22A6, A8, A1, A2, and A3, though IC_50_s were above 70 µM [39]. Our screen to search for drug transporters that might be involved in the intracellular accumulation of afatinib identified for the first time SLC16A1 as a potential candidate. We not only demonstrated that its expression was increased in 22Rv1 clones stably expressing PXR, but also showed that a specific pharmacological inhibitor of SLC16A1 reduced, at least in part, PXR-mediated sensitization to afatinib, with this effect being accompanied by a reduction in its accumulation in cells. 

*SLC16A1* (*MCT1*) belongs to the proton-coupled monocarboxylate transporters family involved in the transport of lactate, pyruvate, and other monocarboxylates [40]. In cancer, SLC16A1 has a role in lactate shuttling between cells, depending on their demand [41]. SLC16A1 is overexpressed and associated with poor prognosis in various types of cancers [42], including prostate cancer where its expression is independent of androgen stimulation [43]. SLC16A1 is also involved in the transport of various drugs. It is involved in XP13512 (gabapentin enacarbil) absorption and explains the better bioavailability of this prodrug in comparison with gabapentin that is used for the treatment of epilepsy and postherpetic neuralgia [44]. SLC16A1 is necessary and sufficient for the uptake of the anti-cancer drug 3-bromopyruvate into tumor cells and its overexpression re-sensitizes 3-bromopyruvate-resistant cells to the drug in xenograft models [45]. A recent study by Lopes-Coelho et al. further confirms that its expression mediates the transport of 3-bromopyruvate in acute myeloid leukemia cell lines and that SLC16A1 could be used as a predictive biomarker of response to this treatment [46]. 

Our results demonstrate for the first time the functional role of SLC16A1 in the transport of afatinib in prostate cancer cells as pharmacological blockage of the transporter by the specific inhibitor BAY-8002 could prevent, at least partially, the sensitization of 22Rv1 cells stably expressing PXR to this kinase inhibitor. Even if afatinib has not been described as a good substrate of this transporter, we cannot exclude that PXR-mediated intracellular accumulation of afatinib may also be due to the induction of other influx pumps that are expressed in prostate cancer such as SLCO1B3 [47]. Since afatinib has been described to inhibit the activity of the ABCB1 transporter in lung cancer cells [48], it is also possible that inhibition of afatinib efflux by may be involved. However, this is unlikely as verapamil did not have any impact on the effect of afatinib on 22Rv1 cell response regardless of the expression of PXR (Figure S8). As evidenced by the binding of PXR to a binding site within a 2000 bp region upstream of the transcription starting site of *SLC16A1* promoter region, our results suggest that SLC16A1 is a PXR target gene. Unexpectedly, binding of PXR to SLC16A1 promoter in the presence of SR12813 did not translate into a significant increase in SLC16A1 mRNA levels in 22Rv1 clones in which PXR was ectopically expressed. This could be attributed to a marginal effect of the agonist due to a high basal activity of PXR and a level of PXR expression that may be sufficient to sustain its activity, as it was already reported [23,49]. Alternatively, this could also be due to a PXR-mediated negative feedback loop attenuating the expression of SLC16A1, as it was demonstrated for the CYP3A4 target gene in HuH7 liver cancer cells overexpressing PXR [50]. However, one cannot exclude that SLC16A1 expression could also be modulated by direct interactions between PXR and other cofactors. For instance, it was previously shown that interaction of PXR with AhR could regulate the basal level of CYP3A4 expression and its activation by PXR agonists both in vitro and in vivo [51,52,53]. It was also demonstrated that both AhR and PXR could regulate the expression of UGT1A1 by binding to different promotor regions of this PXR target gene [54]. It is also possible that PXR could modulate SLC16A1 levels independently of its binding to the SLC16A1 promoter region. Further studies are required to investigate these potential cross-talks in the regulation of SLC16A1 expression by PXR.

## 5. Conclusions

Our results confirm that PXR is significantly expressed in tumor samples from prostate cancer patients and show that its expression is more frequently detected in advanced stages of the disease. We identified for the first time a PXR-mediated role of *SLC16A1* in the transport of afatinib and the sensitivity of prostate cancer cells to this ErBb family blocker. Our study further strengthens the implication of ADME genes in the clinical outcome of various cancers [55] and highlights the importance of PXR as a potential biomarker of response to anticancer treatments in prostate cancers, in particular kinase inhibitors that are still the object of many clinical trials for castration-resistant tumors.

## Figures and Tables

**Table 1:** Patients and tissues characteristics.

Groups	CLC (*n* = 449)	CRPC (*n* = 48)	Metastases (*n* = 28) Bone *n* = 15 Lymph Node *n* = 13
Age y, median (range)	63 (46–74)	72 (56–86)	66 (51–77)
PSA (ng/mL)	10.1 (1.5–23)	12.5 (0.2–285)	12.4 (3.1–4894)
pTNM			
pT2	315	NA	NA
pT3	134		
ISUP group			
1	141	NA	NA
2	148		
3	137		
4–5	23		

CLC: hormone-naïve clinically-localized cancer; CRPC: castration resistant prostate cancer; PSA: Prostate-Specific Antigen; pTNM: pathology Tumor Node Metastasis; ISUP: International Society of Urological Pathology; y: years; NA: not applicable.

**Table 2:** Sequences of the primers used in this study.

*ABCA12*	sense 5′- AGAACAGGCACTGCAAATGAAT antisense 5′-ATGGGAATGGCCAGCAACTT-3′;
*ABCA2*	sense 5′-AGAACGTGACGCTCAAACGC, antisense 5′-GCGATTGCATGACAGGCAGG-3′;
*ABCA5*	sense 5′-TGGAATGGACCCCTGTTCTCG, antisense 5′-CGGTAGCCGATCCCCCATTTA-3′;
*ABCA9*	sense 5′-TGTTTTCTTATGATGCAAGGGCA, antisense 5′-AGGTCTTTCACACAACTTCCTTCA-3′;
*ABCB1*	sense 5′-GTCGGACCACCATTGTGATAG-3′; antisense 5′-CATTTCCTGCTGTCTGCATTGTG-3′,
*ABCC1*	sense 5′-GAATGGCATGCTGGTGACGG-3′; antisense 5′-AGCTTGACCTGCCCTGTCTG-3′;
*ABCC10*	sense 5′-TGCCACCATCCGAGACAACA-3′; antisense 5′-CCTGGTAGACAGCACGAGCA-3′;
*ABCD3*	sense 5′-TCGGCCTGCACGGTAAGAAA-3′; antisense 5′-TCGAGACACCAGCATAACAGCA-3′;
*ABCD4*	sense 5′-CGGAGCCTGCTGCTTTCTAC-3′; antisense 5′-GACCCCGCTGAAAATGGGGA-3′;
*ABCF1*	sense 5′-TCAGGATCAGAGTGAGGAAGAGGA-3′; antisense 5′-AGAGCAGCGAATTTATTTTGAGGC-3′;
*CYP3A4*	sense 5′-GCCTGGTGCTCCTCTATCTA-3′; antisense 5′-GGCTGTTGACCATCATAAAAG-3′;
*GAPDH*	Sense 5′-AATTGAGCCCGCAGCCTCCC-3′; antisense 5′-CCAGGCGCCCAATACGACCA-3′
*HPRT*	sense 5′-CTGACCTGCTGGATTACA-3′; antisense 5′-GCGACCTTGACCATCTTT-3′
*RPL0*	sense 5′-CAGATGGATCAGCCAAGAAGG-3′; antisense 5′-ATCAACGGGTACAAACGAGTC-3′
*SLC15A2*	sense 5′-AGTCCTTGGGTGCCTTACCA-3′; antisense 5′-AGCTCCCTGCATTGATGGACA-3′;
*SLC16A1*	sense 5′-TTAAGGCGGCCCTGTTGAGA-3′; antisense 5′-TCCAATTACCACTGCCCAGC-3′
*SLC16A3*	sense 5′-GGAGGTGAGGCGGAACCAAC-3′; antisense 5′-TCCAGGCTGTGTCGCTGTAG-3′;
*SLC19A2*	sense 5′-TTGCTGCAAACCTCAGCATGG-3′; antisense 5′-CCACTGGCCAGGAAAACCAC-3′;
*SLC19A3*	sense 5′-CGGCAAGTGAGCGATTTGGT-3′; antisense 5′-TCTCTGCACTGGTCAGGTTTTT-3′;
*SLC25A13*	sense 5′-GGCACCAGGAAAGATGTTGAAGT-3′; antisense 5′-GCCTCAGCCAAGTTAAAGGGC-3′;
*SLC2A1*	sense 5′-CATGGCGGGTTGTGCCATAC-3′; antisense 5′-AGAAGCCTGCAACGGCAATG,
*SLC31A1*	sense 5′-CAAGTGGCCAAAACCCCTGT, antisense 5′-CTCTGCCCTGAGGCACAACT-3′;
*SLC38A2*	sense 5′-TCCTACCCCACCAAGCAAGC-3′; antisense 5′-ACCCTCCTTCATTGGCAGTCT-3′;
*SLC3A2*	sense 5′-TCCTTCTTGCCGGCTCAACT-3′; antisense 5′-TTGGCACTTACAGCCCCTGG-3′
*SLC7A11*	sense 5′-CTCTGACTGGAGTCCCTGCG-3′; antisense 5′-TGTCTCCCCTTGGGCAGATTG-3′;
*SLC7A6*	sense 5′-TTCATCCGCCTGTGGGTCTC-3′; antisense 5′-TGCCCCACTTGACATAGGCA-3′

## Data Availability

Data are contained within the article and Supplementary Materials.

## Supplementary Materials

The following are available online at https://www.mdpi.com/article/10.3390/cancers13143635/s1, Figure S1: Validation of PXR stable transfection. (A) Western blots showing basal levels of expression of PXR and SLC16A1 in 22Rv1, DU145, and PC3 prostate cancer cell lines and in 22Rv1 clones 10 and 15 stably transfected with hPXR. GAPDH was used as a loading control. (B) PXR immunostaining using paraffin-embedded cell pellets of 22Rv1-pCDNA control clone 3 as a negative control and of 22Rv1 clone 10 and 15 stably expressing hPXR as positive controls, respectively. (C) Induction of PXR activity by SR12813 in 22Rv1 cells stably expressing PXR. Exponentially growing cells were treated with 3 µM of the PXR agonist SR12813 for 24 h and luciferase activity in clone 10 and 15 were quantified. Results are expressed as ratios of luminescence relative to untreated cells. Figure S2: Effects of PXR stable expression on the sensitivity to kinase inhibitors. Clones 10 or/and 15 stably expressing PXR and the control clone (clone 3) were treated with increasing concentrations of the indicated kinase inhibitors for 72 h and growth inhibition was evaluated as described in Figure 3. Figure S3: Effects of stable expression of PXR on the intracellular accumulation of afatinib. (A) 22Rv1 cells stably expressing PXR (clone 10 and 15) and control cells (clone 3) were treated with 1 µM afatinib for 48 h and concentrations of afatinib were measured using mass spectrometry as described in Figure 4. Results are expressed as intracellular/extracellular ratios of drug concentrations normalized to control clone and are the mean ± SEM of 3 independent experiments. *p* < 0.05 (*), *p* < 0.001 (**) as evaluated by Student’s *t*-test. (B) Stability of afatinib in the culture medium. Afatinib was incubated in culture medium for indicated times and concentrations of the drug were measured using mass spectrometry, normalized to concentrations that were measured directly after adding afatinib (0), and plotted as a function of time. Results are the mean ± SEM of three independent measurements. Figure S4: Drug transporter screening. Raw expression of drug transporter genes as measured by the RT^2^ Profiler™ PCR Array Human Drug Transporters from Qiagen according to the manufacturer’s recommendations. mRNA levels of the 86 genes were measured in both 22Rv1 control clone (clone 3) (A) and 22Rv1 clone 10 stably expressing PXR (B). The graphs show the Ct for each of the 84 genes as obtained from the qPCR experiment. Only genes with a Ct < 30 were selected for further validation using an independent set of primers. Figure S5: Effects of BAY-8002 on cell growth. (A) Effects of the SLC16A1 inhibitor BAY-8002 on the growth of 22Rv1 cells stably expressing PXR. Clone 15 was treated with increasing concentrations of BAY-8002 for 72 h and growth inhibition was evaluated as described in Figure 1. Figure S6: SLC16A1 expression in human primary hepatocytes. mRNA levels of SLC16A1 in normal human hepatocytes following 48 h treatment with 50 µM dabrafenib as measured by RT-qPCR. SLC16A1 expression was normalized to HPRT and expressed as fold change relative to untreated cells (NT). Results are the mean ± SEM of 2 independent experiments. Figure S7: Binding of PXR to *SLC16A1* promoter of 22Rv1 control clone 3 or clone 15 stably expressing PXR, in the absence or in the presence of SR12813 (6 µM, 48h) as measured by chromatin immunoprecipitation as described in Figure 7. Figure S8: Effects of verapamil on PXR-mediated sensitization of 22Rv1 cells to afatinib. Clone 15 stably expressing PXR and the control clone (clone 3) were treated with increasing concentrations of afatinib in the absence or in the presence of 10 µM of the ABCB1 inhibitor verapamil for 72 h and growth inhibition was evaluated as described in Figure 3. Figure S9: Uncropped Western blotting figures.
