# Supplementary material for: PXR Modulates the Prostate Cancer Cell Response to Afatinib by Regulating the Expression of the Monocarboxylate Transporter SLC16A1
Source: Cancers (Basel). 2021 Jul 20;13(14):3635. doi: 10.3390/cancers13143635 (PMC8305337; doi:10.3390/cancers13143635)
Supplement: Supplementary file 1 [file cancers-13-03635-s001.zip › cancers-1245299-supplementary.pdf]

Supplementary Materials:

PXR Modulates the Prostate Cancer Cell Response to Afatinib by Regulating the Expression of the Monocarboxylate Transporter SLC16A1

Alice Matheux, Matthieu Gassiot, Gaëlle Fromont, Fanny Leenhardt, Abdelhay Boulahtouf, Eric Fabbrizio, Candice Marchive, Aurélie Garcin, Hanane Agherbi, Eve Combès, Alexandre Evrard, Nadine Houédé, Patrick Balaguer, Céline Gongora, Litaty C. Mbatchi and Philippe Pourquier

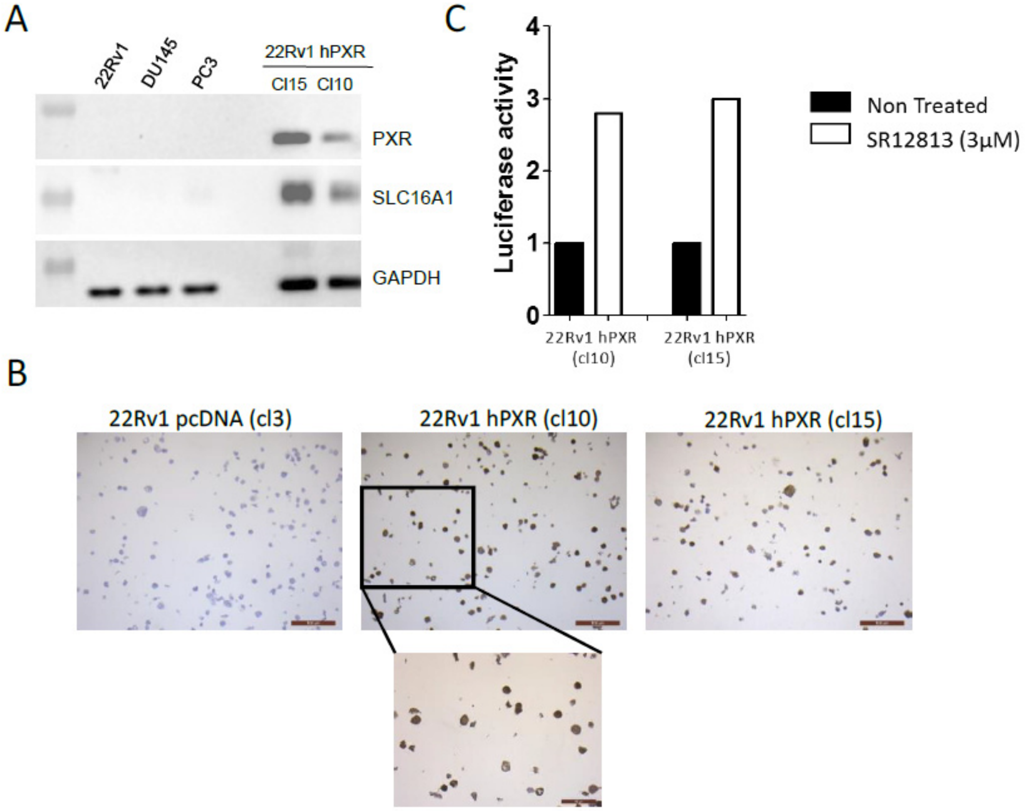

Figure S1. Validation of PXR stable transfection. (A) Western blots showing basal levels of expression of PXR and SLC16A1 in 22Rv1, DU145, and PC3 prostate cancer cell lines and in 22Rv1 clones 10 and 15 stably transfected with hPXR. GAPDH was used as a loading control. (B) PXR immunostaining using paraffin-embedded cell pellets of 22Rv1-pCDNA control clone 3 as a negative control and of 22Rv1 clone 10 and 15 stably expressing hPXR as positive controls, respectively. (C) Induction of PXR activity by SR12813 in 22Rv1 cells stably expressing PXR. Exponentially growing cells were treated with 3 µM of the PXR agonist SR12813 for 24 h and luciferase activity in clone 10 and 15 were quantified. Results are expressed as ratios of luminescence relative to untreated cells.

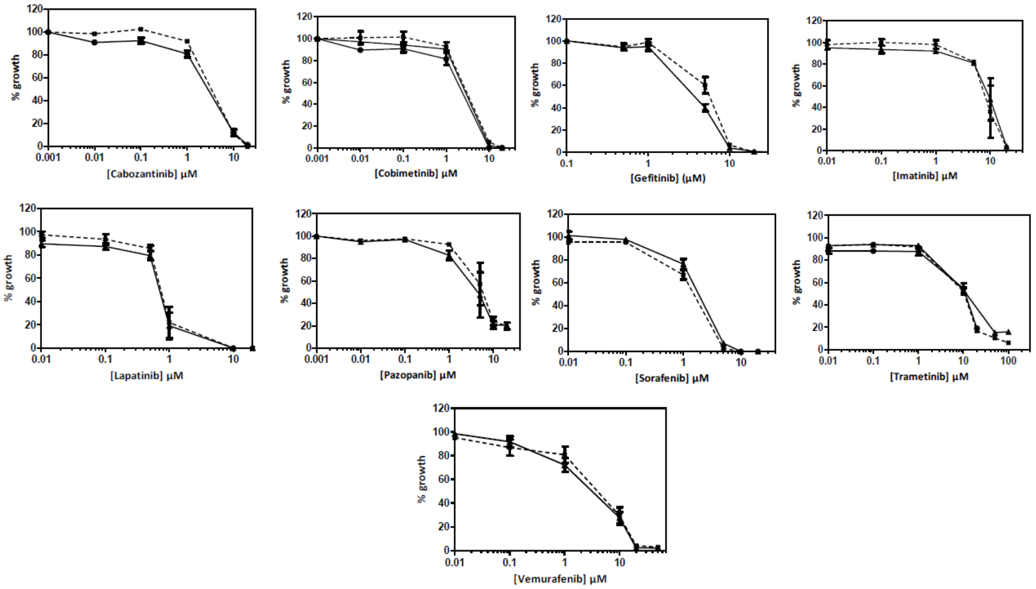

Figure S2. Effects of PXR stable expression on the sensitivity to kinase inhibitors. Clones 10 or/and 15 stably expressing PXR and the control clone (clone 3) were treated with increasing concentrations of the indicated kinase inhibitors for 72 h and growth inhibition was evaluated as described in Figure 3.

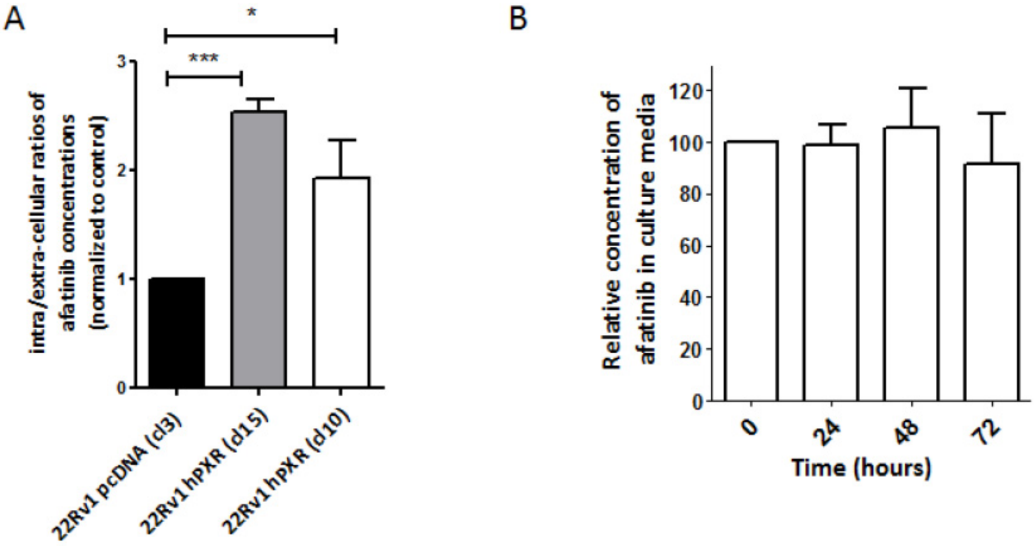

Figure S3. Effects of stable expression of PXR on the intracellular accumulation of afatinib. (A) 22Rv1 cells stably expressing PXR (clone 10 and 15) and control cells (clone 3) were treated with 1 µM afatinib for 48 h and concentrations of afatinib were measured using mass spectrometry as described in Figure 4. Results are expressed as intracellular/extracellular ratios of drug concentrations normalized to control clone and are the mean ±SEM of 3 independent experiments. (*) $p < 0.05$, $p < 0.001$ (**) as evaluated by Student's t-test. (B) Stability of afatinib in the culture medium. Afatinib was incubated in culture medium for indicated times and concentrations of the drug were measured using mass spectrometry, normalized to concentrations that were measured directly after adding afatinib (0), and plotted as a function of time. Results are the mean ±SEM of three independent measurements.

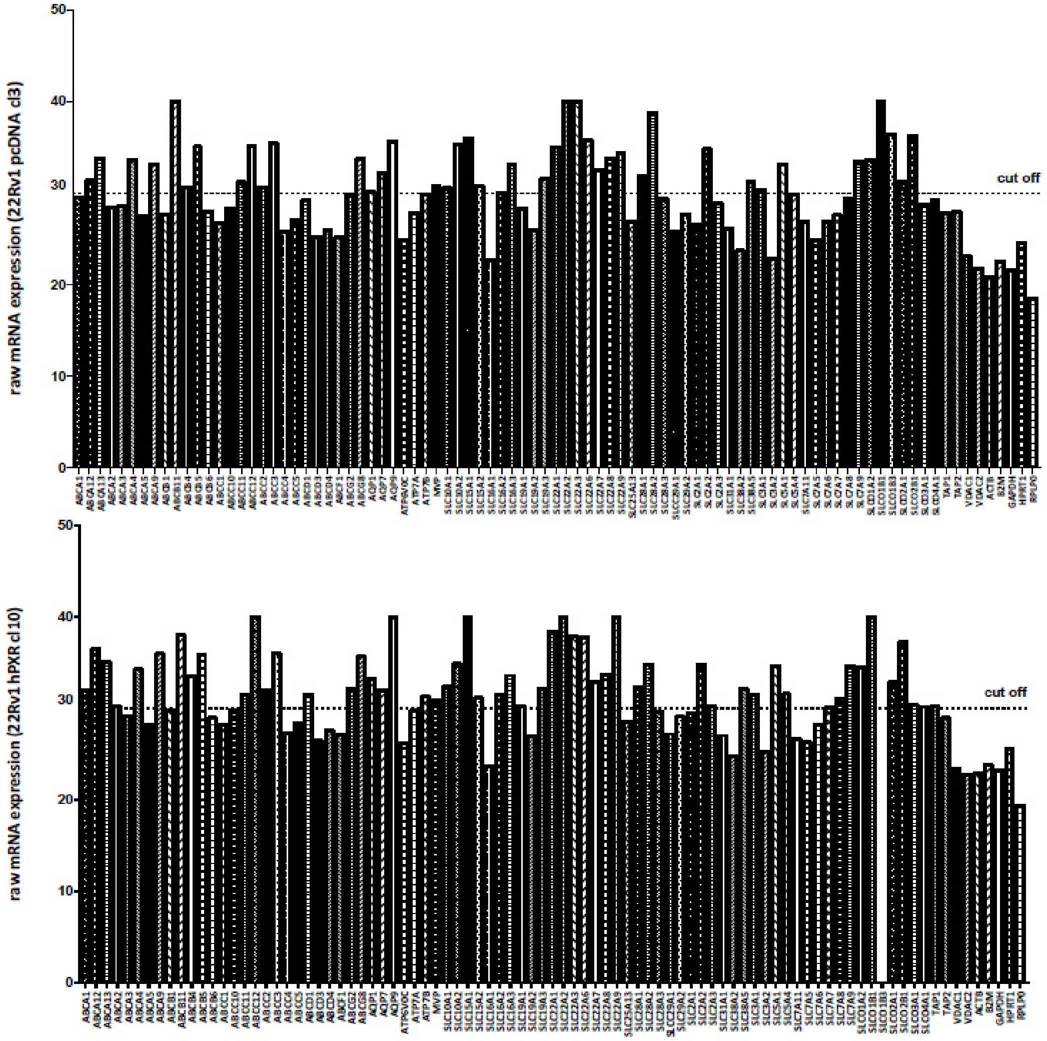

Figure S4. Drug transporter screening. Raw expression of drug transporter genes as measured by the RT² Profiler™ PCR Array Human Drug Transporters from Qiagen according to the manufacturer's recommendations. mRNA levels of the 86 genes were measured in both 22Rv1 control clone (clone 3) (A) and 22Rv1 clone 10 stably expressing PXR (B). The graphs show the Ct for each of the 84 genes as obtained from the qPCR experiment. Only genes with a Ct < 30 were selected for further validation using an independent set of primers.

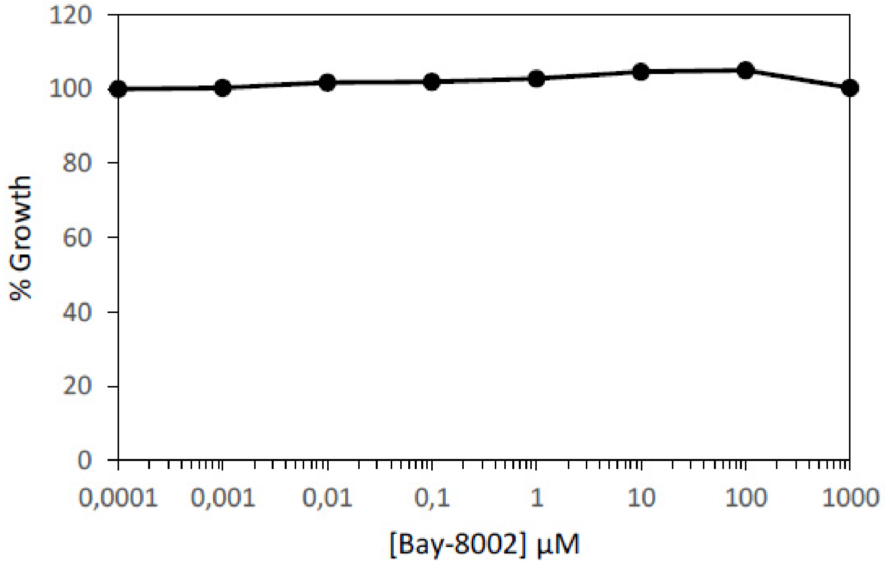

Figure S5. Effects of BAY-8002 on cell growth. (A) Effects of the SLC16A1 inhibitor BAY-8002 on the growth of 22Rv1 cells stably expressing PXR. Clone 15 was treated with increasing concentrations of BAY-8002 for 72 h and growth inhibition was evaluated as described in Figure 1.

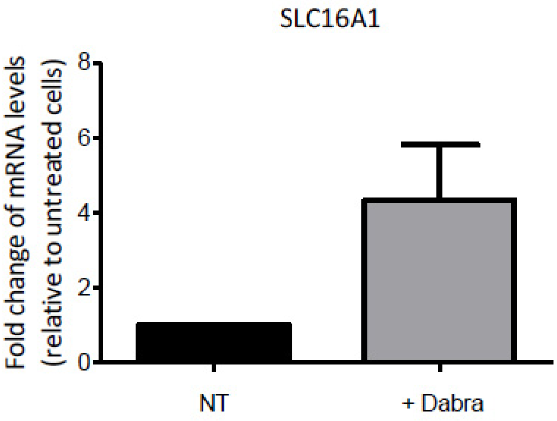

Figure S6. SLC16A1 expression in human primary hepatocytes. mRNA levels of SLC16A1 in normal human hepatocytes following 48 h treatment with 50 μM dabrafenib as measured by RT-qPCR. SLC16A1 expression was normalized to HPRT and expressed as fold change relative to untreated cells (NT). Results are the mean ± SEM of 2 independent experiments.

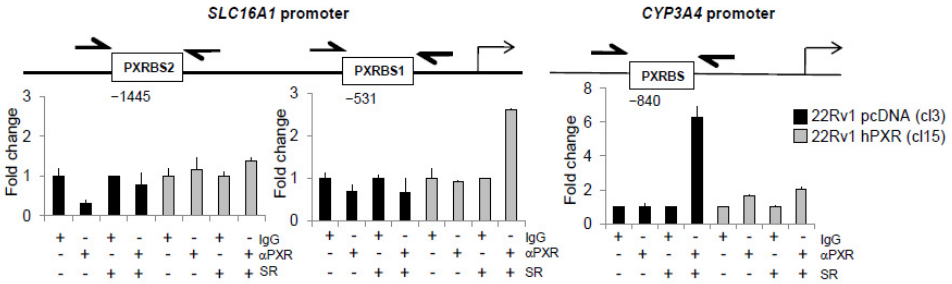

Figure S7. Binding of PXR to *SLC16A1* promoter of 22Rv1 control clone 3 or clone 15 stably expressing PXR, in the absence or in the presence of SR12813 (6 µM, 48h) as measured by chromatin immunoprecipitation as described in Figure 7.

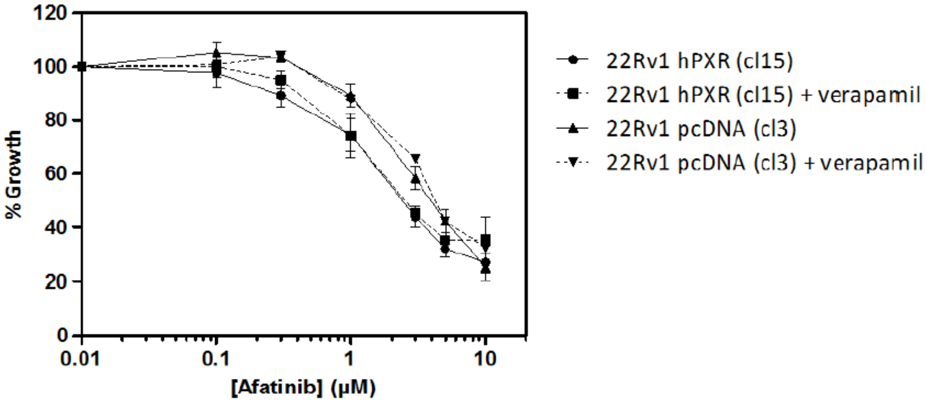

Figure S8. Effects of verapamil on PXR-mediated sensitization of 22Rv1 cells to afatinib. Clone 15 stably expressing PXR and the control clone (clone 3) were treated with increasing concentrations of afatinib in the absence or in the presence of 10 µM of the ABCB1 inhibitor verapamil for 72 h and growth inhibition was evaluated as described in Figure 3.

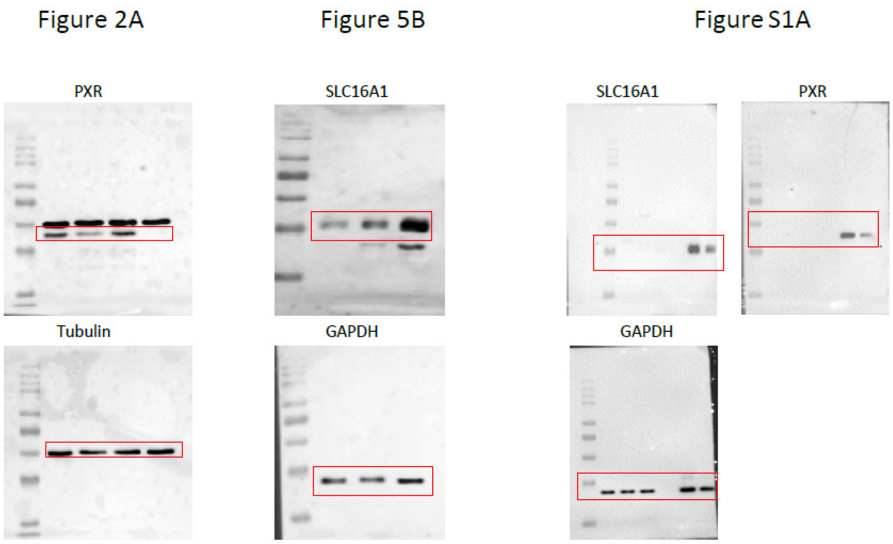

Figure S9. Uncropped Western blotting figures.
